# Therapeutic enhancement of S-1 with CPT-11 through down-regulation of thymidylate synthase in bladder cancer

**DOI:** 10.1002/cam4.95

**Published:** 2013-06-10

**Authors:** Hiroki Ide, Eiji Kikuchi, Masanori Hasegawa, Seiya Hattori, Yota Yasumizu, Akira Miyajima, Mototsugu Oya

**Affiliations:** 1 Department of Urology, Keio University School of MedicineTokyo, Japan

**Keywords:** 5-Fluorouracil, irinotecan, S-1, thymidylate synthase, urothelial carcinoma

## Abstract

Thymidylate synthase (TS), a target enzyme of 5-fluorouracil (5-FU), is significantly associated with prognosis in various cancers. Recently, it has been reported that S-1, a novel 5-FU-based agent has an effect on bladder cancer. However, in cells with high TS level, S-1 did not have significant effects. Therefore, we examined whether down-regulation of TS enhanced effects of S-1 in them. First, we measured TS level in an aggressive bladder cancer cell line, KU-19-19 by enzyme-linked immunosorbent assay (ELISA) and evaluated its sensitivity to 5-FU using a small interfering RNA (siRNA) for TS. Next, we measured TS mRNA after exposure to various agents. Finally, we evaluated enhancement of cytotoxicity of S-1 by CPT-11 (7-ethyl-10-[4-(1-piperidino)-1-piperidino]carbonyloxycamptothecin) which down-regulated TS in in vivo study. The median TS and dihydropyrimidine dehydrogenase (DPD) level was 53.3 ng/mg and 80.3 ng/mg in KU-19-19 cells, respectively. The 5-FU treatment in KU-19-19 cells transfected with siRNA for TS gene (TYMS) inhibited cell growth more significantly than that for nontargeting control. Down-regulation of TS was observed after exposure to SN-38 (7-ethyl-10-hydroxycamptothecin) in a dose-dependent manner. The combination treatment of 5-FU and SN-38 significantly inhibited cell growth, as compared to the single treatment. Meanwhile, in cells transfected with siRNA for TYMS, neither an additive nor a synergistic effect was observed. Also, combined S-1 and CPT-11 dramatically inhibited tumor growth, compared to S-1 or CPT-11 alone in in vivo study. In conclusion, CPT-11 down-regulated TS level and enhanced the effect of S-1. Thus, the combination therapy with S-1 and CPT-11 might be a novel modality for bladder cancer, even with high TS level.

This study confirmed that thymidylate synthase (TS) level in an aggressive human bladder cancer cell line, KU-19-19, was relatively higher than that in other cancer and presented that irinotecan (CPT-11) could down-regulate TS. Finally, the combination therapy with S-1 and CPT-11 resulted in significant tumor growth inhibition through down-regulation of TS in KU-19-19. Thus, combined S-1 and CPT-11 might be a novel treatment in bladder cancer, even with high TS.

## Introduction

Urothelial carcinoma (UC) occurs in the urinary tract, which includes bladder, upper urinary tract, and urethra, and approximately 90% of bladder tumors are UC. The prognosis of bladder cancer patients with locally advanced or lymph node metastasis remains poor, with a median survival of approximately 12 months 1–2. Systemic chemotherapy is the current modality that provides the potential for survival in those with advanced or metastatic bladder cancer. Cisplatin (CDDP)-based chemotherapy, which is the only effective regimen for bladder cancer, has a short-term therapeutic effect against metastatic bladder cancer with a response rate of about 50%; however, the rate of longer survival after receiving systemic chemotherapy is low, with a 5-year survival rate of only 13–15% 3,4. Thus, a novel chemotherapeutic regimen for treating a highly aggressive bladder cancer is needed.

Thymidylate synthase (TS) is the key enzyme that catalyzes the methylation of deoxyuridine monophosphate to deoxythymodine monophosphate, which is an important step in the process of DNA synthesis and a target enzyme of 5-fluorouracil (5-FU) 6–7. Recently, it was reported that TS was significantly associated with prognosis in patients with bladder cancer. Furthermore, we recently showed that a high TS level was an independent predictor of disease-specific survival in patients with upper tract urothelial carcinoma (UTUC) 8. Thus, an agent that can down-regulate the level of TS is important and may enhance the cytotoxic activity of 5-FU-related chemotherapeutic agents in patients with bladder cancer and UTUC. In addition, a recent study demonstrated that TS elevation was associated with resistance to CDDP in lung cancer 9. Therefore, the combination treatment of a 5-FU-related agent with one which can down-regulate the TS level might have potential benefits for patients with CDDP resistance.

S-1 is an oral 5-FU-based antitumor drug containing 5-chloro-2,4-dihydropyrimidine (CDHP; gimeracil) and has been used clinically in various cancers such as of the stomach, colon, lung, and pancreas. CDHP competitively inhibits dihydropyrimidine dehydrogenase (DPD), the enzyme that degrades the majority of 5-FU, approximately 180 times more effectively than uracil, which is a component of Tegafur-Uracil (UFT), and it was reported that S-1 had a significantly higher antitumor effect than UFT in various cancers 10. Recently, we demonstrated that S-1 had a stronger growth inhibitory effect in tumors with a higher level of DPD activity than UFT in a bladder cancer model in vivo. However, in patients with a high level of TS, a single treatment of S-1 did not result in significant tumor growth inhibition 8.

Irinotecan (7-ethyl-10-[4-(1-piperidino)-1-piperidino]carbonyloxycamptothecin, CPT-11) is a synthetically designed analogue of camptothecin that inhibits DNA topoisomerase. It is a water-soluble prodrug that is converted into the more active metabolite 7-ethyl-10-hydroxycamptothecin (SN-38) by carboxyesterase enzymes in the body 11. CPT-11 has been shown to be an active agent in various cancers such as colorectal cancer 12. Also, in advanced bladder cancer, a recent study demonstrated that CPT-11 in combination with gemcitabine (GEM) was an effective treatment 13. Furthermore, CPT-11 could have an enhanced effect on the therapeutic activity of S-1 through its down-regulating TS activity in colon cancer 14. On the other hand, in UC, little is known about how CPT-11 may down-regulate the level of TS and enhance the antitumor effect of S-1. In fact, to the best of our knowledge, there has never been a report that showed the efficacy of S-1 in combination with CPT-11 in UC.

In this study, using a highly aggressive UC cell line, KU-19-19, which was established in our laboratory, (1) we examined the association between TS level and the sensitivity to 5-FU using a small interfering RNA (siRNA) that specifically targets TS and (2) measured the level of TS after exposure to various antitumor agents to identify which agents could down-regulate the enzyme. Finally, (3) we evaluated enhancement of the antitumor activity of S-1 by CPT-11 which can down-regulate the level of TS and measured TS levels in treated tumors in an animal model.

## Material and Methods

### Cell lines

An aggressive human bladder cancer cell line, KU-19-19, was used in this study. KU-19-19, a cytokine-producing cell line, was established in our laboratory from invasive UC of the bladder 15 and used in various in vitro and in vivo studies as a bladder cancer model. KU-19-19 cells were cultured at 37°C in RPMI 1640, a recommended cell culture medium, supplemented with 10% heat-inactivated fetal bovine serum, 100 μg/mL streptomycin (Life Technologies, Inc., Grand Island, NY), and 100 IU/mL penicillin (Life Technologies, Inc.).

### Chemicals

Synthesis of 5-FU was done by Wako Pure Chemical Industries (Osaka, Japan) and SN-38 and CPT-11 were supplied by Yakult Honsha Co., Ltd. (Tokyo, Japan). S-1 was obtained from Taiho Pharmaceutical Co., Ltd. (Tokyo, Japan). CDDP, GEM, and mitomycin C (MMC) were purchased from Wako Pure Chemical Industries, Ltd. (Osaka, Japan). 5-FU, CDDP, GEM, and MMC were dissolved in culture medium to prepare 100 μg/mL solutions and subsequently diluted in culture medium. S-1 was dissolved in distilled water with 0.5% hydroxypropylmethylcellulose (HPMC). SN-38 was dissolved in DMSO to prepare a 1 mg/mL solution and subsequently diluted in culture medium to a final DMSO concentration of less than 0.1%. CPT-11 was dissolved in saline solution by sonication and warming.

### Real-time quantitative PCR

The cells were lysed with RNAiso reagent (Takara Bio Inc., Shiga, Japan) according to the manufacturer's protocol for total RNA extraction. RNA was quantitated by the ratio of absorbance at 260/280 nm. Reverse transcription of RNA to cDNA was conducted using a High Capacity cDNA Archive Kit (Applied Biosystems, Tokyo, Japan). Next, real-time polymerase chain reaction (RT-PCR) was carried out in a final volume of 20 μL containing cDNA template, TS or GAPDH primers (Applied Biosystems, Tokyo, Japan), TaqMan^®^ Universal PCR Master Mix (Applied Biosystems, Tokyo, Japan), and DNase RNAase free water, using the StepOne real-time PCR system (Applied Biosystems, Tokyo, Japan) according to the manufacturer's protocol. Cycling conditions were 50°C for 10 min, 95°C for 10 min, and then 40 cycles at 95°C for 15 sec and 60°C for 1 min. The data were then quantified using the comparative *C*_t_ method for relative gene expression compared with GAPDH as endogenous control. The primers and TaqMan probe sets for TS (TYMS) (Hs00426591_m1) and human GAPDH endogenous control (Hs99999905_m1) were purchased from Applied Biosystems.

### Small interfering RNA

Two predesigned siRNAs for TYMS and nontargeting control (NTC) siRNA (AllStars Negative Control siRNA) were obtained from Invitrogen Co. (Tokyo, Japan). KU-19-19 cells (1.5 × 10^5^ per well) were cultured in antibiotic-free medium overnight at 37°C in 5% CO_2_ and then transfected with the siRNA for TYMS or NTC, using Lipofectamine Max (Invitrogen Co., Tokyo, Japan). Forty-eight hours later, the transfected cells were washed and used for subsequent experiments.

### Cell growth assay

Briefly, 2 × 10^4^ cells were seeded in each well of 96-well plates and allowed to grow overnight. The cells were then treated with various concentrations of 5-FU, CDDP, GEM, MMC, and SN-38 for 72 h. Cells transfected with or without siRNA for TYMS or NTC were treated with 5-FU in combination with or without SN-38 for 72 h. Cytotoxicity was determined using WST-1; 4-[3-(4-lodophenyl)-2-(4-nitrophenyl)-2H-5-tetrazolio]-1, 3-benzene disulfonate (Takara Bio Inc, Shiga, Japan). The absorbance value of each well was determined at 450 nm with a 650 nm reference beam using a microplate reader (Bio-Rad Laboratories, Inc., Tokyo, Japan).

### Enzyme-linked immunosorbent assay

Each sample (1.0 × 10^8^ cells or xenograft tumors) was homogenized in a 10-fold volume of sample weight of the diluting solution (20 mmol/L PBS, which contained 0.05% Tween 20) and centrifuged at 105,000 ×*g*, 4°C for 1 h. The supernatant (100 μL) was then dispensed onto an antihuman TS or DPD polyclonal antibody (Solid phase antibody: Mitsubishi Chemical Medience, Tokyo, Japan) immobilized plate and incubated for 1 h at room temperature. After the wells were washed four times with PBS, 100 μL aliquots of horseradish peroxidase were conjugated to antihuman TS or DPD polyclonal antibody (Label antibody: Mitsubishi Chemical Medience, Tokyo, Japan). After the wells were washed four times with PBS, 100 μL aliquots of 0.1 mol/L acetate buffer (pH 5.5; color-developing solution) containing 3 mg/mL orthphenylenediamine and 0.75 mmol/L hydrogen peroxide were added, followed by incubation for 30 min in the dark. Finally, 100 μL aliquots of 1 mol/L sulfuric acid were added to terminate the reaction and the measurements were conducted with the measuring wavelength of an enzyme-linked immunosorbent assay (ELISA) plate reader set at 490 nm.

### Treatment in vivo

All of the procedures involving animals and their care in this study were approved by the Animal Care Committee of Keio University in accordance with institutional and Japanese government guidelines for animal experiments. Female BALB/c-*nu/nu* mice were obtained from Sankyo Lab Service Co. (Tokyo, Japan). KU-19-19 cells (2 × 10^6^) were implanted s.c. into the flanks of the nude mouse and then, mice were randomly assigned to one of four groups. Tumors were treated with daily oral administration of S-1 (8.3 mg/kg) and/or weekly i.v. administration of CPT-11 (33 mg/kg). The combination group was treated with daily S-1 (8.3 mg/kg) and weekly i.v. administration of CPT-11 (33 mg/kg). Control animals only received vehicle by gavage. Each experimental group consisted of 10 mice. The animals were carefully monitored, and tumor size and body weight were measured every 4 days. Twenty-four days after tumor cell implantation, the mice were sacrificed and the tumors were collected. Tumor volume was calculated according to the formula *a*^2^ × *b* × 0.52, where *a* and *b* are the smallest and largest diameters, respectively.

### Statistical analysis

The levels of TS and DPD are presented as the mean ± SD. The other data are presented as the mean ± SE. The difference between two groups in the in vitro study and in the animal model was assessed with the Mann–Whitney *U*-test. The level of statistical significance was set at *P *< 0.05. Combination index (CI) analysis provided qualitative measure of the extent of drug interaction. A CI of less than, equal to, and more than 1 indicated synergy, additivity, and antagonism, respectively 16. These analyses were performed with an SPSS Version 16.0 statistical software package (SPSS Corporation, Tokyo, Japan).

## Results

### The levels of TS and DPD and effects of siRNA targeting TS on the sensitivity to 5-FU in KU-19-19 cells

In KU-19-19 cells, the median TS and DPD protein levels were 53.3 and 80.3 ng/mg, respectively, and the mean ± SD of TS and DPD protein levels were 53.1 ± 12.0 and 85.3 ± 40.8 ng/mg, respectively.

**Figure 1:**
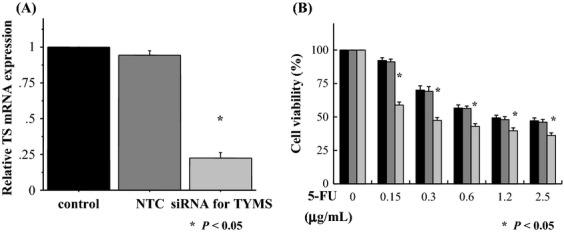
Effects of siRNA-specific for TS on the sensitivity to 5-FU in KU-19-19 cells. (A) Relative expression of TS mRNA in KU-19-19 cells treated with or without siRNA for TYMS. Nontargeting control (NTC) siRNA was also used as a negative control. (B) Effects of siRNA for TYMS on the sensitivity of KU-19-19 cells to 5-FU. KU-19-19 cells transfected with siRNA for TYMS or NTC were treated with various concentrations of 5-FU. The cell viabilities were measured by WST assay; bars, +SE. *P < 0.05 compared to controls.

Transfection of siRNA for TYMS reduced the mRNA level of TS to 20% in KU-19-19 cells (*P* < 0.05) compared to vehicle control, whereas no difference in the TS mRNA expression was observed by transfection with NTC (Fig. 1A). The 5-FU treatment (0.15 μg/mL or higher) in KU-19-19 cells transfected with siRNA for TYMS resulted in significantly higher levels of cell growth inhibition compared to that in KU-19-19 cells transfected with siRNA for NTC or vehicle control (Fig. 1B). Also, it was shown that the 50% inhibitory concentration (IC_50_) of 5-FU in KU-19-19 cells transfected with siRNA targeting for TYMS (0.26 ± 0.06 μg/mL) was significantly lower than that of vehicle control (1.17 ± 0.08 μg/mL) or that in cells transfected with siRNA for NTC (1.05 ± 0.07 μg/mL).

### TS levels after exposure to anticancer drugs

**Figure 2:**
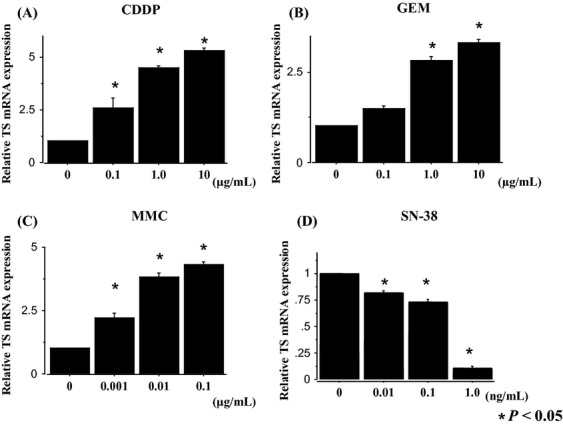
TS levels after exposure to various anticancer drugs. Expression of TS mRNA in KU-19-19 cells exposed to various concentrations of CDDP (cisplatin) (A), GEM (gemcitabine) (B), MMC (mitomycin C), (C) and SN-38 (D); bars, +SE. *P < 0.05 compared to controls.

Figure 2 shows the relative TS mRNA levels after exposure to CDDP, GEM, MMC, and SN-38 for 72 h. Up-regulation of TS was observed after exposure to CDDP, GEM, and MMC in a dose-dependent manner. Meanwhile, down-regulation of TS was observed with SN-38 in a dose-dependent manner.

### Cytotoxic effects of 5-FU and SN-38 treatment in KU-19-19 cells treated with or without siRNA for TYMS

**Figure 3:**
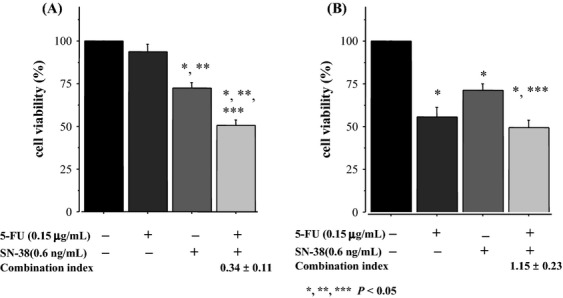
Cytotoxic effects of the combination of 5-FU with SN-38 in KU-19-19 cells with or without siRNA-specific targeting TS. The mean cell viability values (relative to control) of the treatment with 5-FU alone (0.15 μg/mL), SN-38 alone (0.6 ng/mL), and combined 5-FU and SN-38 in KU-19-19 cells without (A) and with siRNA for TYMS (B); bars, +SE. *P < 0.05 compared to controls. **P < 0.05 compared to 5-FU groups. ***P < 0.05 compared to SN-38 groups.

The combination treatment of 5-FU (0.15 μg/mL) and SN-38 (0.6 ng/mL) significantly inhibited cell growth (cell viability; 50.4 ± 1.8%) compared to vehicle control to a greater degree than that 5-FU (93.9 ± 2.2%) or SN-38 (72.6 ± 1.2%) treatment alone (Fig. 3A).

In KU-19-19 cells treated with siRNA for TYMS, the mean cell viability values relative to control in cells treated with 5-FU alone (0.15 μg/mL), SN-38 alone (0.6 ng/mL), and combined 5-FU and SN-38 were 55.5 ± 4.2%, 71.3 ± 1.6%, and 49.4 ± 3.0%, respectively (Fig. 3B). The combination index values of 5-FU (0.15 μg/mL) and SN-38 (0.6 ng/mL) combination treatment in KU-19-19 cells without and with siRNA for TYMS were 0.34 ± 0.11 and 1.15 ± 0.23, respectively; therefore, a synergistic effect of the combination treatment was observed in KU-19-19 cells without any transfection of siRNA for TYMS, while neither an additive nor a synergistic effect was observed in cells transfected with siRNA for TYMS.

### TS levels in KU-19-19 cells treated with 5-FU and SN-38

**Figure 4:**
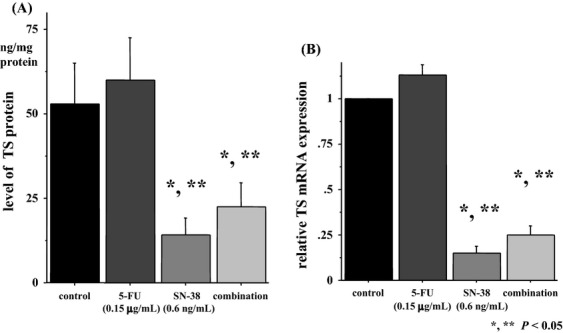
TS levels in KU-19-19 cells treated with 5-FU and/or SN-38. The TS protein level (A) and relative TS mRNA expression (B) in KU-19-19 cells treated with 5-FU alone (0.15 μg/mL), SN-38 alone (0.6 ng/mL), and combined 5-FU and SN-38; bars, +SE. *P < 0.05 compared to controls. **P < 0.05 compared to 5-FU groups.

Figure 4A and B present the levels of TS protein and relative TS mRNA expression after exposure to 5-FU, SN-38, and combined 5-FU and SN-38, respectively for 72 h. The TS protein level (14.3 ± 4.9 ng/mg protein) and relative TS mRNA expression after exposure to SN-38 (0.15 ± 0.04) was significantly lower than those in control (53.1 ± 12.0 ng/mg protein, 1.00 ± 0.00, respectively). Furthermore, the TS protein level (22.7 ± 6.8 ng/mg protein) and relative TS mRNA expression (0.25 ± 0.05) of combination treatment of 5-FU with SN-38 was significantly lower than those of control. In contrast, the TS protein level and relative TS mRNA expression were up-regulated after exposure to 5-FU (60.2 ± 12.2 ng/mg protein, 1.13 ± 0.06, respectively) compared to control.

### In vivo effect of S-1 and CPT-11 on KU-19-19 tumors in nude mice

**Figure 5:**
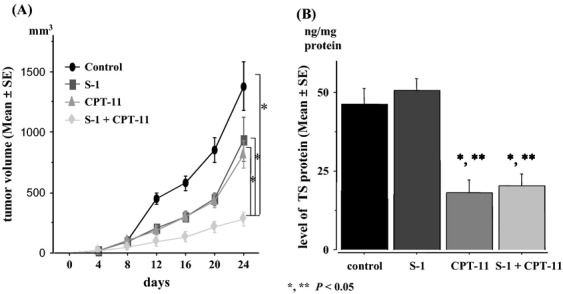
Antitumor effect of S-1 in combination with CPT-11 in KU-19-19 xenograft model. (A) Treatment in vivo. KU-19-19 cells (2 × 10^6^) were implanted s.c. into the flank of nude mice. Tumors were treated with daily oral administration of S-1 (8.3 mg/kg) and weekly i.v. administration of CPT-11 (33 mg/kg). The combination group was treated with S-1 (8.3 mg/kg) followed by weekly i.v. administration of CPT-11 (33 mg/kg). Control animals only received vehicle by gavage. Mean tumor volumes in mm^3^ are shown; bars, ±SE. *P < 0.05. (B) The TS protein levels in tumors treated with S-1 and/or CPT-11; bars, ±SE. *P < 0.05 compared to controls, **P < 0.05 compared to S-1 groups.

In vivo experiments revealed significant decreases in mean tumor volume in mice treated with S-1 and CPT-11 (282 ± 59 mm^3^) compared to those treated with medium vehicle alone (1382 ± 202 mm^3^), S-1 alone (941 ± 184 mm^3^), or CPT-11 alone (814 ± 113 mm^3^) in the KU-19-19 tumor model 24 days after tumor implantation (*P* < 0.05 for all) (Fig. 5A). Meanwhile, there were no significant differences between the mean tumor volume in control mice and mice treated with S-1 alone or CPT-11 alone.

Figure 5B presents the TS protein levels in tumors in mice treated with S-1, CPT-11, and combined S-1 and CPT-11. The mean ± SE of the protein level of TS in tumors treated with CPT-11 (18.1 ± 8.0 ng/mg protein) was significantly lower than that in tumors treated with vehicle control (46.1 ± 10.0 ng/mg protein). Furthermore, the mean ± SE of the TS protein level in tumors treated with the combination treatment of S-1 and CPT-11 (20.2 ± 7.6 ng/mg protein) was significantly lower than that in tumors treated with S-1 alone (50.7 ± 7.2 ng/mg protein).

## Discussion

Bladder cancer is one of the most aggressive epithelial tumors and is characterized by a high rate of early systemic dissemination. Most patients with advanced or metastatic bladder cancer are treated with systemic chemotherapy including CDDP; however, the response rates remain inadequate. Furthermore, there are few effective regimens for bladder cancer patients with CDDP-resistance. Therefore, establishment of a new therapeutic option would be a very important task for the management of advanced or metastatic bladder cancer.

In this study, we first measured the TS level in a KU-19-19 cell, which is a human bladder cancer cell line. In KU-19-19 cells, the median TS and DPD protein levels were 53.3 and 80.3 ng/mg, respectively, and the mean ± SD of TS and DPD protein levels were 53.1 ± 12.0 and 85.3 ± 40.8 ng/mg, respectively. According to a previous report, median TS and DPD protein levels in various cancers including head and neck, gastric, colorectal, breast, lung, and pancreatic cancers were 22.1 and 134.8 ng/mg, respectively, and the mean ± SD of TS and DPD protein levels were 39.9 ± 53.8 and 159.8 ± 116.3 ng/mg, respectively. Although these data were not compared directly, the TS level in KU-19-19 cells is relatively higher than that in other cancers reported, while the DPD level in KU-19-19 cells is lower than that in other cancers reported 17.

We next examined the association between the level of TS and sensitivity to 5-FU using siRNA for TYMS in order to down-regulate the TS expression in KU-19-19 cells. In this study, down-regulation of the TS level by the siRNA resulted in significant enhancement of the cytotoxicity to 5-FU. In previous reports, the TS levels in gastric and colon cancers were also significantly correlated with 5-FU sensitivity, with high TS levels resulting in low sensitivity to 5-FU 18,19. We have previously demonstrated an association between 5-FU sensitivity and down-regulation of the TS level with siRNA in the bladder cancer cell line, UMUC-3 cells 8. Thus, the result of this study in KU-19-19 cells was in accordance with that of the previous study in UMUC-3 cells.

Secondly, we measured the level of TS after exposure to various chemotherapeutic agents in order to examine whether these agents could affect the TS level. Our study showed that most agents, including GEM, CDDP, and MMC, which have been used as chemotherapeutic agents for bladder cancer in general, up-regulated the TS level. However, only SN-38 down-regulated the TS level in a dose-dependent manner. In general, the TS level was up-regulated after exposure to most agents, as most cancer cells gained resistance to chemotherapeutic agents. According to a previous report in lung cancer cells exposed to CDDP, the level of TS was significantly up-regulated and these cells developed resistance to CDDP. Moreover, this study demonstrated that the up-regulation of TS was associated with the phosphorylation of mitogen-activated protein kinase kinase 1/2-extracellular signal-regulated kinase 1/2 9.

Thirdly, we evaluated the cytotoxicity of 5-FU in combination with SN-38. There was significant growth inhibition in cells treated with combined 5-FU and SN-38 compared to cells treated with SN-38 or 5-FU alone. In addition, based on the CI index, a synergistic effect was observed in cells treated with the combination treatment. Interestingly, in cells in which the level of TS was down-regulated using siRNA for TYMS, neither an additive nor a synergistic effect of the combination treatment was observed. This result revealed that down-regulation of the TS level might be associated with the enhancement of 5-FU in our in vitro study.

Finally, the combination treatment of S-1 with CPT-11 was evaluated in the KU-19-19 subcutaneous tumor model. The results demonstrated that significant inhibition of tumor growth was observed in tumors treated with S-1 in combination with CPT-11 as compared to tumors treated with S-1 or CPT-11 alone. Furthermore, our study showed that the TS protein level in tumors treated with the S-1 and CPT-11 combination treatment was significantly lower than that in tumors treated with S-1 alone. These results showed that the therapeutic effects of S-1 enhanced by CPT-11 were highly associated with the down-regulation of TS levels in KU-19-19 tumors in our in vivo study.

In conclusion, this was the first study demonstrating the efficacy of S-1 in combination with CPT-11 in bladder cancer. Recently, there have been several studies on the effects of S-1 in bladder cancer, in particular with a high level of DPD on the basis of the fact that S-1 has the strongest DPD inhibitor among 5-FU-related agents 8–21. However, an effective strategy for bladder cancer with a high level of TS has never been established. In this study, we demonstrated that CPT-11 could enhance the cytotoxic effects of S-1 in aggressive bladder cancer cells with a higher level of TS expression. Therefore, combination treatment consisting of S-1 and CPT-11 could be a novel chemotherapeutic regimen in bladder cancer, even with higher levels of TS and DPD.
